# Clinical factors associated with invasive pulmonary aspergillosis in patients with severe fever with thrombocytopenia syndrome: analysis of a 6-year clinical experience

**DOI:** 10.3389/fmicb.2024.1448710

**Published:** 2024-09-12

**Authors:** Huan Wang, Miao Luo, David Fisher, Khrystyna Pronyuk, Erkin Musabaev, Hien Nguyen Thi Thu, Pian Ye, Lei Zhao

**Affiliations:** ^1^Department of Infectious Diseases, Union Hospital, Tongji Medical College, Huazhong University of Science and Technology, Wuhan, China; ^2^Department of Medical Biosciences, Faculty of Natural Sciences, University of the Western Cape, Cape Town, South Africa; ^3^Infectious Diseases Department, O. Bogomolets National Medical University, Kyiv, Ukraine; ^4^The Research Institute of Virology, Ministry of Health, Tashkent, Uzbekistan; ^5^Hai Phong University of Medicine and Pharmacy, Hai Phong, Vietnam

**Keywords:** invasive pulmonary aspergillosis, severe fever with thrombocytopenia syndrome, risk score, prognosis, clinical experience

## Abstract

**Background:**

Invasive pulmonary aspergillosis (IPA) typically occurs in immunocompromised individuals. Severe fever with thrombocytopenia syndrome (SFTS) patients are typically characterized by fever, thrombocytopenia, and leukopenia. These patients typically present with dysregulation of cellular and humoral immunity, which may predispose them to IPA. Our study aimed to identify risk factors for SFTS-associated invasive pulmonary aspergillosis (SAPA) and evaluate its associated prognostic impact.

**Methods:**

We conducted a cohort study between January 2017 and December 2022 in a tertiary hospital in Wuhan City, China. All SFTS patients hospitalized in our department who formally consented were divided into a SAPA group and a non-SAPA group according to whether they were coinfected with aspergillosis or not. The independent risk factors for the SAPA group were determined by multivariate logistic regression. Receiver operating characteristic (ROC) analysis was used to assess the statistical value of parameters to predict SAPA patients. The survival analysis was carried out using the Kaplan–Meier (KM) method.

**Results:**

Of the 269 hospitalized SFTS patients enrolled in the study, 118 (43.87%) cases were diagnosed with SAPA with an average age of 65.71 ± 9.7 years. Multivariate logistic regression analysis revealed that age, neurological complications, serum severe fever with thrombocytopenia syndrome virus (SFTSV) RNA loads, the white blood cell (WBC) count, platelet (PLT) count, albumin (ALB) and globulin (GLB) concentrations, and cardiac troponin I (cTNI) were complementary risk factors for the development of IPA in SFTS patients. The risk score is calculated as 5 times age, plus 6 times neurological complications, plus 10 times RNA (log), plus 5 times WBC, minus 5 times PLT, minus 5 times ALB, plus 5 times GLB, and plus 6 times cTNI. ROC curve analysis showed that the area under the receiver operating characteristic (AUROC) curve represented a risk score of 0.837 (95% *CI*: 0.789–0.885, *p* < 0.001) for predicting IPA in SFTS patients. The average length of hospitalization in the SAPA group was more prolonged than non-SAPA. SAPA and non-SAPA groups had significantly different mortality rates: 25.42% (SAPA) and 3.97% (non-SAPA) (*p* < 0.05).

**Conclusion:**

SFTS patients with IPA have high morbidity and mortality. Early monitoring of neurological complications, SFTSV RNA loads, WBC, PLT, ALB, GLB, and cTNI in SFTS patients may be useful in predicting the occurrence of IPA.

## Introduction

Severe fever with thrombocytopenia syndrome (SFTS) is an emerging infectious disease, caused by *Dabie bandavirus* of the Bandavirus genus of the Phenuiviridae family, associated with a high fatality rate of 5.2–16.2%, which was first reported in China in 2009, and has been reported in Japan, South Korea, Vietnam, and many other countries (Yu et al., 2011; Huang et al., 2021; Li et al., 2018; McMullan et al., 2012). The main clinical manifestations are summarized as fever, thrombocytopenia, leukopenia, dyspnea, digestive symptoms, hemorrhage, and neurological symptoms. The severity of these symptoms and multi-organs involvement could result in death (Li et al., 2018). There are no effective vaccines that can efficiently potentiate the immune system to SFTSV or specific therapeutic agents against SFTS. However, several studies have reported the therapeutic benefit of favipiravir (Yuan et al., 2021; Suemori et al., 2021; Li et al., 2021). A previous study has reported an 8–30% incidence of coinfections among SFTS patients, which exacerbate mortality, with fungal coinfections being particularly pervasive (Zhang et al., 2022). Reports in the literature have indicated that SFTSV infections appear to effectively avoid or delay the triggering of the intracellular innate immune responses in humans, leading to the attenuation of cellular and humoral immune responses, which in turn, increases susceptibility to coinfections (Song et al., 2018). Contrary studies have reported that cytokine storms and macrophage activation syndrome resulting from SFTSV infection are associated with multiple organ dysfunction syndrome (MODS), which in turn may be linked with secondary infections (He et al., 2021; Kim et al., 2017).

IPA mainly occurs in immunocompromised individuals, most notably those patients (a) presenting with neutropenia for more than 10 days; (b) with hematologic malignancy; (c) as recipients of allogeneic stem cell or solid organ transplant; (d) on prolonged treatment of corticosteroids; (e) on treatment with recognized T or B-cell immunosuppressants; or (f) with inherited severe immunodeficiency (Donnelly et al., 2020). Critically ill SFTS patients have been confirmed to have immune dysfunction together with leukopenia and thrombocytopenia, which might further contribute to the immunosuppression and the development of IPA. Secondary infections are closely associated with severe type SFTS individuals, with the most prevalent strains as *Aspergillus fumigatus*, *Aspergillus flavus*, *Candida*, *Klebsiella pneumonia*, and *Escherichia coli* (with the respiratory tract being the most common infection site) (Zhang et al., 2022), among which Aspergillosis is the most frequent pathogenic infection. Several studies reported IPA increased susceptibility in critical SFTS patients contributing to increased mortality (Bae et al., 2019; Xu et al., 2021; Yao et al., 2024). Bae et al. (2019) reported that 20% (9/45) of the patients with SFTS developed IPA. Xu et al. (2021) study reported that 31.9% (29/91) of hospitalized patients had complications of IPA, with in-hospital mortality up to 22%, while the Yao et al. (2024) study found that the mortality rate of SAPA was 26.6%. However, few studies on severe fever with SFTS have reported the contributory risk factors for IPA. Therefore, accurate and timely identification of the risk factors of IPA in SFTS is essential for early identification and improved survival of SFTS patients.

In this study, we analyzed a 6-year clinical data set of SFTS patients from Wuhan Union Hospital to identify factors for IPA in SFTS patients and to evaluate its impact on outcome, providing prognostic evidence for early diagnosis and timely treatment.

## Materials and methods

### Study population

The complete medical records of adult patients with confirmed SFTS, hospitalized between 1 January 2017 and 31 December 2022 in the Department of Infectious Diseases at Wuhan Union Hospital, were included in this study, with anonymity being guaranteed. The SFTS individuals included in the study must have met a minimum of one of the following criteria: (a) the positive detection of SFTSV RNA in the patient’s serum; (b) the detection of IgM against SFTSV or a four-fold increase of serum IgG against SFTSV during the convalescent phase; and (c) the detection of SFTSV genome by next-generation sequencing (Seo et al., 2021). Exclusion criteria were patients who (a) were immunosuppressed or complicated with IPA before the infection of SFTSV; (b) had acute or active viral coinfections besides SFTSV; (c) were discharged within 48 h after admission; or (d) did not have chest computed tomography (CT) images and a galactomannan (GM) test. The following data were recorded: demographic characteristics, comorbidities, initial symptoms and clinical signs, biological and microbiological parameters, radiography findings, course during hospitalization, treatments, and prognosis. The study was approved by the human ethics committee of Tongji Medical College (2023-S093). Although written consent by patients was waived by the ethics committee due to the retrospective nature of the study, all data were anonymized to protect the personal information of the patients.

As immunocompromised SFTS patients were routinely excluded, patients with probable SAPA were diagnosed based on the criteria published by the European Organization for the Research and Treatment of Cancer/Mycosis Study Group (EORTC/MSG), clinical features and mycologic evidence of aspergillosis. The clinical features included evidence on presence of one of the following CT manifestations: dense, well-circumscribed lesions with or without a halo sign, air crescent sign, cavity, wedge-shaped and segmental or lobar consolidation. Mycological evidence included:① a positive culture from sputum, bronchoalveolar lavage (BAL), bronchial brush; ② galactomannan (GM) index in BAL ≥1 or GM index in serum ≥0.5. (Donnelly et al., 2020). The radiological chest computed CT images of SAPA patients were analyzed by two independent radiologists.

### Statistical analysis

Statistical analysis was performed using R software (version 3.5.2). Continuous variables were expressed as mean ± standard deviation (SD), and categorical variables were summarized as counts (percentage). The chi-square test or Fisher’s exact test was used for categorical variables. The *t*-test was used when continuous data were normally distributed, while the Mann–Whitney U-test was used for continuous data that was not normally distributed. All tests of significance were two-sided, and a *p*-value of <0.05 was considered statistically significant. ROC curve analysis was performed for the independent risk factors. Continuous variables were converted into dichotomous variables according to the area under the curve (AUC) using the best cutoff value. Univariate analysis with dichotomous variables was performed, followed by stepwise logistic regression to screen for risk factors. The logistic regression model was established based on these factors and their regression coefficients. In addition, AUC and the best cutoff value with the corresponding sensitivity and specificity were calculated to assess the value of parameters to predict SAPA patients. Survival analysis comparison between the SAPA and non-SAPA groups was performed using the KM curve based on the log-rank test.

## Results

### Clinical characteristics

From January 2017 and December 2022, 269 hospitalized SFTS patients admitted to Wuhan Union Hospital with complete medical records were included in the study. Most of the patients lived in the Dabie Mountain area and worked as farmers. Patient baseline demographic characteristics, underlying medical conditions, clinical manifestations, laboratory findings at admission, treatment, and outcome parameters between the SAPA and non-SAPA patients are summarized in Table 1. Briefly, 118 (43.87%) cases were diagnosed with SAPA, consisting of 70 men and 48 women, with an average age of 65.71 (± 8.42) years, which was older than the non-SAPA group (61.03 ± 10.08, *p < 0.05*). There were 97 male individuals and 54 female individuals in the non-SAPA group, with no significant difference compared to the SAPA group.

**Table 1:** Comparisons of clinical and laboratory characteristics between SAPA and non-SAPA patients.

Characteristics	Total (*n* = 269)	Non-SAPA group (*n* = 151)	SAPA group (*n* = 118)	*p*-value
**Demographics**				
Age, years	63.08 ± 9.70	61.03 ± 10.08	65.71 ± 8.52	<0.001
Male	167 (62.08)	97 (64.24)	70 (59.32)	0.41
Mortality	36 (13.38)	6 (3.97)	30 (25.42)	<0.001
**Underlying conditions**				
Smoking	44 (16.36)	21 (13.91)	23 (19.49)	0.219
Drinking	31 (11.52)	13 (8.61)	18 (15.25)	0.09
Hypertension	88 (32.71)	43 (28.48)	45 (38.14)	0.094
T2DM	73 (27.14)	33 (21.85)	40 (33.90)	0.027
COPD	11 (4.09)	5 (3.31)	6 (5.08)	0.675
**Clinical manifestations**				
Tmax	38.95 ± 0.60	38.95 ± 0.63	38.95 ± 0.56	0.919
Neurological symptoms	97 (36.06)	32 (21.19)	65 (55.08)	<0.001
Bleeding	59 (21.93)	31 (20.53)	28 (23.73)	0.529
Muscle fibrillation	37 (13.75)	13 (8.61)	24 (20.34)	0.006
**Laboratory parameters**				
SFTSV log	3.40 ± 1.30	2.99 ± 1.16	3.92 ± 1.29	<0.001
WBC	3.55 ± 2.86	3.32 ± 2.50	3.85 ± 3.25	0.13
Hb	125.49 ± 18.41	126.00 ± 17.78	124.84 ± 19.23	0.609
PLT	49.06 ± 29.04	53.68 ± 32.95	43.15 ± 21.85	0.003
Lymphocyte	0.81 ± 0.62	0.80 ± 0.54	0.82 ± 0.72	0.732
Monocyte	0.28 ± 0.38	0.22 ± 0.21	0.36 ± 0.50	0.004
Neutrophil	2.43 ± 2.40	2.27 ± 2.19	2.62 ± 2.64	0.234
ALT	126.87 ± 146.66	116.47 ± 125.91	140.19 ± 169.18	0.189
AST	380.65 ± 551.13	306.16 ± 398.58	475.97 ± 689.75	0.012
Scr	81.53 ± 43.87	72.08 ± 24.05	93.63 ± 58.34	<0.001
LDH	1218.92 ± 1129.40	943.07 ± 703.29	1571.92 ± 1436.58	<0.001
CK	1438.87 ± 3061.35	1441.01 ± 3793.89	1436.14 ± 1736.04	0.99
cTni	527.94 ± 1610.60	279.44 ± 1135.77	845.93 ± 2026.43	0.007
CK-MB	6.16 ± 9.05	5.54 ± 8.33	6.96 ± 9.88	0.201
AMY	219.73 ± 177.60	185.70 ± 127.11	263.27 ± 219.39	<0.001
Lipase	430.74 ± 456.81	349.09 ± 337.25	535.22 ± 559.06	0.002
FBG	7.56 ± 3.25	6.74 ± 2.18	8.61 ± 4.01	<0.001
ALB	31.24 ± 4.49	32.25 ± 4.42	29.94 ± 4.26	<0.001
GLB	26.49 ± 4.64	25.83 ± 4.10	27.34 ± 5.15	0.008
Na	135.40 ± 4.63	135.09 ± 3.99	135.80 ± 5.32	0.21
K	3.67 ± 0.50	3.59 ± 0.46	3.77 ± 0.53	0.003
Ca	1.93 ± 0.14	1.94 ± 0.13	1.92 ± 0.15	0.169
APTT	57.78 ± 18.61	54.05 ± 14.35	62.55 ± 22.10	<0.001
FIB	3.02 ± 6.00	2.62 ± 0.64	3.52 ± 9.03	0.22
INR	1.02 ± 0.14	1.01 ± 0.12	1.04 ± 0.17	0.12
CRP	12.63 ± 21.02	9.91 ± 21.44	16.11 ± 20.02	0.016
PCT	1.07 ± 2.83	0.66 ± 1.27	1.59 ± 3.97	0.016
**Treatment**				
PLT transfusion	175 (65.06)	83 (54.97)	92 (77.97)	<0.001
IVIG	149 (55.39)	74 (49.01)	75 (63.56)	0.017
Glucocorticoids	87 (32.34)	38 (25.17)	49 (41.53)	0.004
GSF	125 (46.47)	75 (49.67)	50 (42.37)	0.234
Antifungal Therapy	171 (63.57)	93 (61.59)	78 (66.10)	0.445

SFTS, severe fever with thrombocytopenia syndrome; SAPA, SFTS-associated invasive pulmonary aspergillosis; T2DM, type 2 diabetes; COPD, chronic obstructive pulmonary disease; T, temperature; SFTSV log, SFTS virus logarithm; WBC, white blood cell; Hb, hemoglobin; PLT, platelet; ALT, alanine aminotransferase; AST, aspartate aminotransferase; Scr, serum creatinine; LDH, lactate dehydrogenase; CK, creatine kinase; cTni, cardiac Troponin I; AMY, amylase; FBG, fasting blood glucose; ALB, albumin; GLB, globulin; Na, sodium; K, potassium; Ca, calcium; APTT, activated partial thromboplastin time; FIB, fibrinogen; INR, international normalized ratio; CRP, C-reactive protein; PCT, procalcitonin; IVIG, intravenous immunoglobulin; GSF, granulocyte stimulating factor.

Reference value ranges: SFTS viral load < 100 TCID50/ml; WBC 3.5–9.5 × 10^9^/L, Hb 115–50 g/L, PLT 125–350 × 10^9^/L, Lymphocyte 1.1–3.2 × 10^9^/L, Monocyte 0.1–0.6 × 10^9^/L, Neutrophil 1.8–6.3 × 109/L, ALT 7–40 U/L, AST 13–35 U/L, Scr 44–106 U/L, LDH 109–245 U/L, CK 26–140 U/L, cTni 0–26.2 ng/L, CK-MB 0–6.6 ng/mL, AMY 20–125 U/L, Lipase 8–78 U/L, FBG 3.9–6.1 mmol/L, ALB 35–55 g/L, GLB 20–30 g/L, Na 136–145 mmol/L, K 3.5–5.2 mmol/L, Ca 2.03–2.54 mmol/L, APTT 28–43.5 s, FIB 2.0–4.0 g/L, INR 0.8–1.2, CRP 0–8 mg/L, PCT 0–0.5 ng/mL.

As for underlying conditions, there were no differences in the incidence of smoking or alcohol consumption between the two groups (Table 1). The proportion of patients with hypertension, diabetes, and chronic obstructive pulmonary disease (COPD) was higher in the SAPA group (38.14, 33.9, and 5.08%, respectively) than in the non-SAPA group (28.48, 21.85, 3.31%, respectively). Regarding to clinical manifestations, all patients presented with a high fever (38.95 ± 0.6°C), while at the initial visit to the hospital, the SAPA group showed a significantly higher incidence of neurological complications (55.08%) and muscle tremor (20.34%) compared to the non-SAPA group (*p < 0.05*). However, there was no significant difference in the incidence of gastrointestinal, nasal, oral, or vaginal bleeding between the groups.

Laboratory findings (Table 1) at admission showed that all patients had reduced PLT counts (49.06 ± 29.04×10^9^/L). The levels of blood SFTSV RNA loads, aspartate aminotransferase (AST), serum creatinine (Scr), GLB, lactate dehydrogenase (LDH), cTNI, amylase (AMY), lipase, fasting blood glucose (FBG), activated partial thromboplastin time (APTT), C-reactive protein (CRP), and procalcitonin (PCT) in the SAPA group were higher than those in the non-SAPA group (Table 1; *p < 0.05*). In contrast, the levels of PLT and ALB in the SAPA group were lower than those in the non-SAPA group (Table 1; *p < 0.05*).

The frequency of PLT transfusion, corticosteroid, and the use of intravenous immunoglobulin (IVIG) was higher in the SAPA group than those in the non-SAPA group (*p < 0.05*), but no significant difference in antifungal and granulocyte stimulating factor (GSF) treatment was found between the groups.

### Risk factors for IPA in SFTS patients

**Figure 1:**
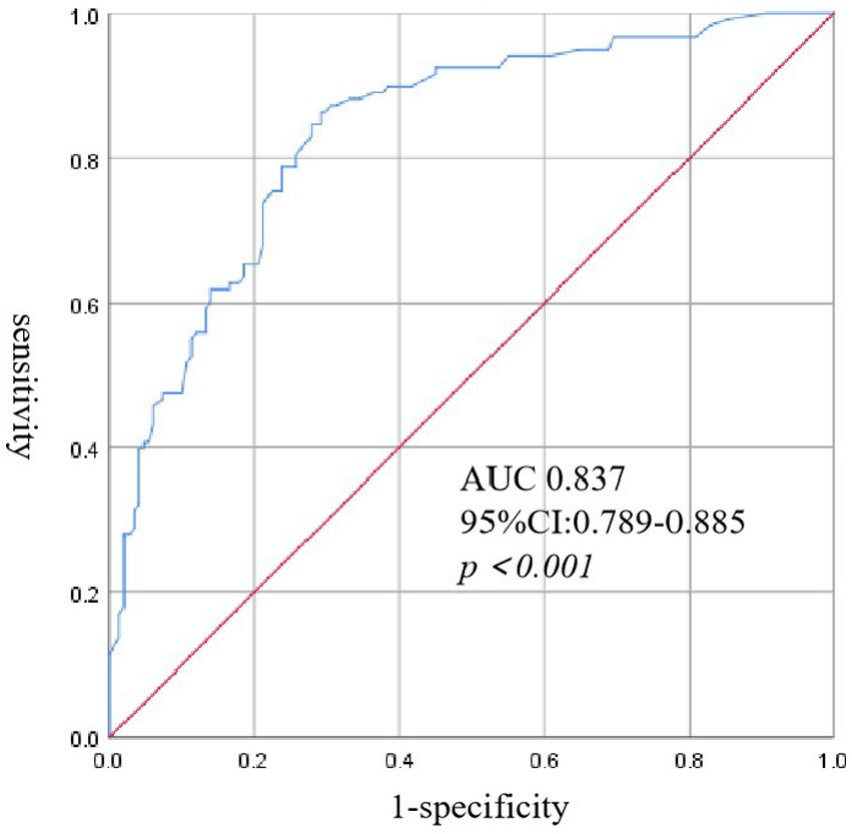
Receiver operating characteristic (ROC) curve analysis of risk score for IPA in patients with SFTS.

Univariate analysis showed that age, neurological complications, muscle tremor, diabetes, PLT transfusion, corticosteroid and IVIG use, as well as high-level SFTSV RNA loads, CRP, PCT, WBC, ALT, AST, ALB, GLB, Scr, CK, LDH, cTNI, AMY, lipase, FBG, APTT, and serum potassium, and low-level PLT were the risk factors for the development of IPA in SFTS (Table 2). Multivariate logistic regression analysis revealed that age, neurological complications, serum SFTSV RNA, WBC, PLT, ALB, GLB, and cTNI were the independent risk factors for the development of IPA in SFTS patients. The risk score was calculated using the following equation: (5 × [Age]) + (6 × [neurological complications]) + (10 × [RNA(log)]) + (5 × [WBC]) – (5 × [PLT]) – (5 × [ALB]) + (5 × [GLB]) + (6 × [cTNI]). ROC curve analysis (Figure 1) showed that the area under the ROC curve (AUC) produced a risk score of 0.837 (95% *CI*: 0.789–0.885, *p < 0.05*) for predicting IPA in SFTS patients.

**Table 2:** Univariate and multivariate logistic regression analysis of risk factors for the development of SAPA.

	Univariate analysis			Multivariate analysis				
Variables	β	OR (95% CI)	*p*-value	β	OR (95% CI)	*p*-value	Weighted points	Modified points
Age_63.5	0.93	2.53 (1.54–4.15)	<0.001	0.824	2.278 (1.245, 4.168)	0.008	5.42	5
Sex	0.21	1.23 (0.75–2.02)	0.41					
Smoking	0.4	1.50 (0.78–2.87)	0.221					
Drinking	0.65	1.91 (0.90–4.08)	0.094					
Hypertension	0.44	1.55 (0.93–2.59)	0.095					
T2DM	0.61	1.83 (1.07–3.15)	0.028					
COPD	0.45	1.56 (0.47–5.26)	0.469					
T_38.6	0.41	1.50 (0.86–2.62)	0.153					
Neurological complications	1.52	4.56 (2.68–7.77)	<0.001	0.846	2.33 (1.24, 4.378)	0.009	5.57	6
Bleeding	0.19	1.20 (0.67–2.15)	0.53					
Muscle fibrillation	1	2.71 (1.31–5.59)	0.007					
RNA_log_3.5	1.48	4.38 (2.62–7.33)	<0.001	1.520	4.574 (2.415,8.662)	0.001	10.0	10
WBC_3.52	0.62	1.85 (1.12–3.07)	0.017	0.696	2.005 (1.05,3.83)	0.035	4.58	5
HB_132	−0.38	0.68 (0.41–1.13)	0.137					
PLT_29	−1.06	0.35 (0.19–0.63)	<0.001	−0.806	0.447 (0.216,0.925)	0.03	5.30	5
Lymphocyte_0.49	−0.46	0.63 (0.38–1.04)	0.07					
Monocyte_0.06	−0.63	0.53 (0.30–0.96)	0.037					
Neutrophil_1.36	0.52	1.69 (1.02–2.77)	0.04					
ALT_91	0.61	1.84 (1.13–2.99)	0.015					
AST_250	1.12	3.07 (1.86–5.07)	<0.001					
Scr_74.0	1	2.71 (1.65–4.48)	<0.001					
CK_407	0.85	2.35 (1.37–4.02)	0.002					
LDH_965	1.26	3.52 (2.13–5.84)	<0.001					
cTni_155.9	1.4	4.04 (2.41–6.79)	<0.001	0.884	2.421 (1.291,4.541)	0.006	5.82	6
AMY_288	0.91	2.49 (1.40–4.42)	0.002					
CK_MB_2.0	0.76	2.15 (1.24–3.70)	0.006					
Lipase	0.9	2.45 (1.44–4.15)	<0.001					
ALB_30.6	−1.04	0.35 (0.21–0.58)	<0.001	−0.783	0.457 (0.244,0.855)	0.014	5.15	5
GLB_28.1	0.72	2.04 (1.23–3.40)	0.006	0.784	2.189 (1.162,4.124)	0.015	5.16	5
FBG_6.0	1.06	2.90 (1.69–4.98)	<0.001					
Na_138.9	0.62	1.85 (0.98–3.51)	0.06					
K_3.86	0.78	2.19 (1.31–3.64)	0.003					
Ca_1.95	−0.43	0.65 (0.40–1.06)	0.087					
APTT_53.8	1.06	2.88 (1.74–4.75)	<0.001					
FIB_2.45	−0.28	0.76 (0.46–1.23)	0.258					
INR_1.07	0.07	1.07 (0.63–1.81)	0.807					
CRP_6.5	1.41	4.09 (2.45–6.83)	<0.001					
PCT_0.81	1.2	3.34 (1.89–5.87)	<0.001					
PLT_transfusion	1.06	2.90 (1.69–4.98)	<0.001					
IVIG	0.6	1.81 (1.11–2.97)	0.018					
Glucocorticoids	0.75	2.11 (1.26–3.55)	0.005					
GSF	−0.29	0.75 (0.46–1.21)	0.234					
Antifungal therapy	0.2	1.22 (0.74–2.01)	0.446					

### IPA infection impact on SFTS outcome

**Figure 2:**
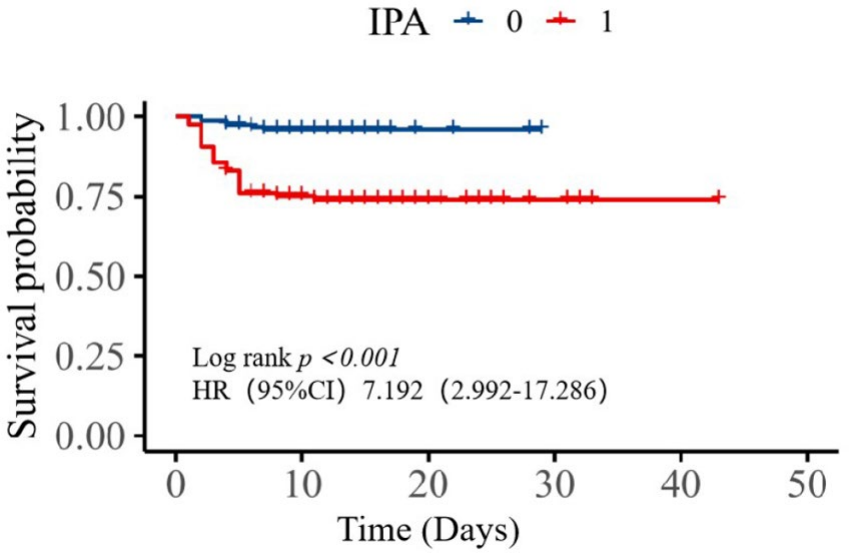
Kaplan–Meier curves for SFTS patients in the IPA and non-IPA groups.

For all patients, the average length of hospitalization in the SAPA group was 12.08 days and 11.09 days for the non-SAPA group with no significant differences (Table 3; *p = 0.185*); in recovering patients, the average length of hospitalization in the SAPA group was 14.97 days and 11.37 days for the non-SAPA group, with a significant difference (p < 0.05). The mortality rate was 25.42% for the SAPA group and 3.97% for the non-SAPA group with a significant difference (*p* < 0.05). Figure 2 shows the comparison of survival curves between the two groups. The non-SAPA group had a higher survival probability than the SAPA group during the hospitalization period (*p* <0.001). Most deaths in the SAPA group occurred in the first week, and the mortality rate decreased after the first week.

**Table 3:** Comparisons of average length of hospitalization between SAPA and non-SAPA patients.

Average length of hospitalization	Non-SAPA group	SAPA group	*p*-value
	Mean (±SD)	Mean (±SD)	
All (151/118)	11.09 ± 4.16	12.08 ± 7.84	0.185
Survival (145/88)	11.37 ± 3.98	14.97 ± 6.92	<0.001

## Discussion

Previous studies have reported the incidence of IPA in SFTS patients ranging from 20 to 39.5% (Bae et al., 2019; Xu et al., 2021; Hu et al., 2021; Song et al., 2023). Our study observed a higher incidence of SFTS patients developing SAPA at 43.87%. The higher incidence of SFTS patients developing SAPA was probably due to mild cases being treated in a nearby local hospital, while more severe cases were referred to our tertiary hospital. The mortality was 25.42% in the SAPA group, which was lower than the studies from Xu et al. (2021), Bae et al. (2019), and Hu et al. (2021) with 31.9, 44.4, and 53.6%, respectively. In the non-SAPA group, our mortality rate was much lower compared to the studies from Song et al. (2023) and Yao et al. (2024), which reported mortality of 27.3 and 26.6%, respectively. A study of 315 patients with influenza who were non-immunocompromised according to EORTC/MSG found 45 (14%) patients with IPA (Schauwvlieghe et al., 2018). These data indicated that SFTS patients with normal immune function would be more prone to Aspergillus infection than influenza and would also be associated with higher mortality. Therefore, accurate and early diagnosis of IPA in SFTS is essential for improving survival of SFTS patients.

Risk factors associated with IPA in non-immunocompromised patients include those who are critically ill, are of advanced age, are diabetics, are treated with short and long courses of glucocorticoids, are treated with broad antibiotic therapy, and have organ dysfunctions (Tudesq et al., 2019). Based on the univariate analysis, our study revealed that critically ill, advanced age, FBG, diabetes, and corticosteroid use were independent risk factors for the development of SAPA. The critically ill included patients with neurological complications, muscle tremors, high-level SFTSV RNA loads, and impairment of multiple organs (cardiovascular, hepatic, pancreatic, and kidney), which was consistent with previous studies for the early prediction of mortality and early diagnosis for IPA (Li et al., 2018; Xu et al., 2021). Based on the multivariate logistic regression analysis, we created a clinical scoring equation that used eight variables (age, neurological complications, serum SFTSV RNA, WBC, PLT, ALB, GLB, and cTNI) to predict the risk of developing SAPA. To the best of our knowledge, this is the first clinical scoring equation that is effective in predicting the risk of developing SAPA. We found that SFTS patients over 63.5 years old, with neurological complications, serum SFTSV RNA loads more than 3,162 IU/mL, WBC more than 3.52 g/L, PLT less than 29 g/L, ALB less than 30.6 g/L, GLB more than 28.1 g/L, and cTNI more than 155.9 ng/L were more likely to develop into IPA. From all variables, serum SFTSV RNA loads were the strongest predictor (10 points), followed by neurological complications and cTNI (6 points), and age, WBC, PLT, ALB, and GLB (5 points each). Our results align well with the previous study by Yao et al. (2024), who found that advanced age, petechiae, hemoptysis, tremors, low ALB levels, prolonged APTT, intensive care unit admission, use of glucocorticoids and IVIG, and prolonged hospital stays were risk factors for SAPA, but not serum SFTSV RNA loads. In comparison to previous studies, Xu et al. (2021) reported wheezing as a non-specific clinical manifestation and an independent predictor for SAPA, while Hu et al. (2021) identified some less easily accessible immune indicators for predicting IPA in SFTS patients. Our study utilized common clinical manifestations and laboratory tests, assigning weights to different indicators to predict SAPA. Thus, early monitoring of neurological complications, SFTSV RNA loads, WBC, PLT, ALB, GLB, and cTNI in SFTS patients may be useful in predicting the occurrence of IPA.

Neurological complications, including apathy, dysphoria, muscular tremors, lethargy, confusion, coma, and convulsions, were common in COVID-19 and severe SFTS infections. These complications have been confirmed in the literature to be associated with increased mortality (Wang et al., 2022), potentially linked to hypoxemia, toxic/metabolic derangements, and systemic inflammation. A previous study from China, using multivariable logistic regression analysis, showed that the incidence of neurological complications was higher in the SAPA group, but with no significant difference (Song et al., 2023). In our study, the incidence of neurological complications was 21.9 and 55.08% between non-SAPA and SAPS groups, respectively (*p<0.001*). The result of multivariable logistic regression analysis further confirmed that neurological complications were an independent predictor for SAPA. The mechanism for why SFTS patients with neurological complications are more likely to develop IPA is unclear. It was hypothesized that patients with neurological complications are more likely to have respiratory depression and difficulty in coughing up sputum, leading to invasive fungal infection. The appearance of infection in patients with neurological complications conforms in part to immunological mechanisms triggered by acute brain injury (Wang et al., 2022; Chamorro et al., 2007). Thus, we should pay more attention to neurological complications, as it is not only associated with critical illness and mortality but also with the development of SAPA.

High serum SFTSV RNA load is associated with neurological complications and mortality (Huang et al., 2021; Wang et al., 2022). Based on the multivariate logistic regression analysis, we also found that high viral load was an independent risk factor for the development of SAPA, which was consistent with Dai et al.’s (2023) study, which showed, using univariate analysis, that high serum viral load showed a significant relationship with SAPA, but when applying multivariate analysis, statistical significance was not achieved. However, other reported studies regarding SAPA did not investigate the relationship between serum viral load and the occurrence of SAPA (Xu et al., 2021; Hu et al., 2021; Song et al., 2023). Previous studies have shown that high serum SFTSV RNA load can induce unbalanced cytokine distribution, which may induce a cytokine storm (Sun et al., 2012). Cytokine storms are a major pathophysiologic mechanism that aggravates leukopenia and thrombocytopenia, which subsequently might result in further immunosuppression, promoting SAPA.

IPA mostly occurs in immunocompromised individuawls, notably those presenting with neutropenia for more than 10 days. In our study, to the contrary, a WBC of more than 3.52 g/L is associated with the development of SAPA. This result is consistent with Xu et al. (2021) and Song et al. (2023), both of whom found that WBC was higher in the SAPA group than in the non-SAPA group (3.1(1.35–5.6) vs. 3(1.8–4.4) and 3.4 ± 2.7 vs. 2.9 ± 1.4, respectively). A possible explanation is that leukopenia and neutropenia are self-restrictive, contrary to what is found in hematological malignancy (Pagano et al., 2017). Another possible reason is that patients may have had prior treatment with colony-stimulating factors in a local hospital or emergency department prior to admission to our Tertiary Hospital. The possible contributing mechanism is a rapid increase in immune cells with the use of colony-stimulating factors, which may induce an immune restructuring syndrome (Dai et al., 2023). In a future study, we will try to investigate the relationship between leukocyte dynamics and SAPA. In addition, PLTs have an impact on the course of invasive fungal infections via immune mediators (Speth et al., 2014). In support of this view, in our study, we found lower levels of PLT in the SAPA group compared with the non-SAPA group, which is consistent with a previous study (Hu et al., 2021).

Short and long courses of glucocorticoids are risk factors associated with IPA in non-immunocompromised patients (Tudesq et al., 2019). In our study, the frequency of corticosteroid use was significantly higher in the SAPA group than in the non-SAPA group. However, multivariable logistic regression analysis did not support it as an independent predictor for SAPA. Previous studies showed that the complications of secondary infections, including IPA, tended to be more frequent in the corticosteroid-treated group, which is consistent with our research (Kawaguchi et al., 2021). Thus, the administration of glucocorticoids to SFTS patients at high risk for invasive Aspergillus infection should be carefully considered. Another finding of our study implies that there was no significant difference in antifungal treatment between the two groups. Because most of the SFTS patients admitted to our hospital were critically ill, prophylactic antifungal medications were administered to all critically ill patients before a definitive diagnosis of IPA was made. In the future, we will initiate prophylactic antifungal therapy for patients with a high risk of IPA.

Previous studies showed that SFTS patients with diabetes did not increase mortality; however, patients with high FBG levels on admission were associated with an increased risk of death regardless of being diabetic (Pan et al., 2024; Ge et al., 2022). Few studies have discussed the effect of blood glucose levels on Aspergillus infections (Xu et al., 2021). In our study, diabetes was a common underlying disease with an incidence of 21.85 and 33.9% in the non-SAPA and SAPS groups, respectively (*p* < 0.05). The FBG levels were 6.74 ± 2.18 mmol/L and 8.61 ± 4.01 mmol/L in the non-SAPA and SAPS groups, respectively (*p* < 0.001), which suggested that patients with high fasting blood plasma glucose levels on admission might be a risk factor for the development of SAPA. Our consideration in this regard is consistent with the study of Song et al. (2023) and Xu et al. (2021). Song’s study recognized uncontrolled diabetes as a risk factor for the development of SAPA, while Xu’s research showed that diabetes was a risk factor for IPA with univariate analysis. However, multivariable logistic regression analysis did not support them as an independent predictor for IPA. Patients with high levels of blood glucose are conducive to the growth of Aspergillus through the inhibition of leukocyte chemotaxis, reduced phagocytosis of phagocytes, and decreased complement production. SFTS infection can cause acute pancreatitis and cytokine storms, which may suppress insulin secretion and exacerbate insulin resistance (Li et al., 2018; Accili, 2021), potentially explaining why critically ill patients presenting with hyperglycemia are more likely to develop invasive Aspergillus infections, leading to poorer survival outcomes. Thus, SFTS patients with diabetes and elevated FBG levels should receive additional attention.

Our study had several limitations. First, this is a single-center retrospective study, and some mild cases were not screened for fungal infections, which may introduce a degree of selection bias. Second, we only assessed the short-term survival outcome of SFTS individuals with IPA, and we could not assess the long-term survival outcome due to the unavailable data. Third, our risk score needs validation from external, prospective, and larger data samples. Fourth, we did not include the treatment effect of IPA in the survival analysis.

## Conclusion

SAPA has high morbidity and mortality. Early monitoring of neurological complications, SFTSV RNA, WBC, PLT, ALB, GLB, and cTNI in SFTS patients may be useful in predicting the occurrence of IPA. Our research holds the potential to help clinicians in the early identification and diagnosis of SFTS patients with IPA, leading to improved strategies for antifungal treatment and patient outcomes.

## Data Availability

The original contributions presented in the study are included in the article/supplementary material, further inquiries can be directed to the corresponding authors.
